# Kawasaki disease shock syndrome with pulmonary involvement in a child: a case report

**DOI:** 10.3389/fimmu.2026.1930430

**Published:** 2026-09-07

**Authors:** Xuhua Xia, Jiwei Zhou, Lan Chen, Lei Zhang

**Affiliations:** Department of Medical General Ward, Children’s Hospital of Chongqing Medical University, National Clinical Research Center for Children and Adolescents’ Health and Diseases, Ministry of Education Key Laboratory of Child Development and Disorders, Chongqing Key Laboratory of Pediatric Metabolism and Inflammatory Diseases, Chongqing, China

**Keywords:** case report, intravenous immunoglobulin, Kawasaki disease, Kawasaki disease shock syndrome, pulmonary involvement

## Abstract

Kawasaki disease (KD) is an acute systemic vasculitis that can affect multiple organs. Kawasaki disease shock syndrome (KDSS) is a severe complication of KD. Pulmonary involvement is uncommon in KD, and there are very few reports of patients with KD who develop KDSS and pulmonary lesions. This report presents the case of a 9-year-old boy who was hospitalized for fever, neck mass and rash, and was subsequently diagnosed with KDSS. During the administration of intravenous immunoglobulin (IVIG), he experienced acute dyspnea, which required urgent invasive mechanical ventilation and bronchoscopy. He was successfully weaned off ventilation shortly and recovered completely. No coronary dilatation or pulmonary fibrosis was detected at the 1-month post-discharge follow-up. This case suggests that KD can trigger rare but severe complications including KDSS and pulmonary involvement. The relationship between pulmonary complications, transfusion-related acute lung injury (TRALI) and transfusion-associated circulatory overload (TACO) remains to be further elucidated. Early aggressive management and comprehensive treatment may improve prognosis.

## Introduction

Kawasaki disease (KD) is a prevalent systemic vasculitis syndrome that primarily affects children younger than 5 years old and is the leading cause of acquired heart disease in the pediatric population (1, 2). The acute-phase vasculitis associated with KD can lead to a range of multisystem complications, including encephalopathy, stroke, retropharyngeal edema, pericarditis, myocarditis, intestinal pseudo-obstruction, gallbladder hydrops, arthritis, and myositis (1). KD shock syndrome (KDSS) is a rare but severe form of the illness in which patients present with more severe laboratory markers of inflammation and greater risk of coronary artery abnormalities, mitral regurgitation, and prolonged myocardial dysfunction (3). Among these systemic clinical manifestations, pulmonary involvement may be diverse and distinctly rare in KD (4–6).

The pulmonary involvement associated with KD shares similar clinical features to those of infectious pneumonia, primarily including fever, cough, wheezing, and dyspnea (7). Notably, respiratory distress is extremely rare among KD patients. While pulmonary involvement is not a common complication of KD, it can present in various forms, including pneumonia, pulmonary nodules, hydropneumothorax, subsegmental atelectasis, pulmonary consolidation and pleural effusion (4, 8–10). Previous research shows that vascular leakage may be a key factor in the pathophysiology of KD (11), and elevated pulmonary vascular permeability is hypothesized to underlie KD-related pulmonary lesions (12).In a cohort, all KD patients with pulmonary involvement were treated with intravenous immunoglobulin (IVIG) and aspirin, resulting in complete resolution of pulmonary symptoms during follow-up (7).

Herein, we report a case of KDSS with pulmonary involvement requiring mechanical ventilation. The patient suddenly developed dyspnea and hypoxemia during IVIG infusion and was immediately transferred to the intensive care unit (ICU).

## Case presentation

A 9-year-old boy was admitted to the inpatient ward due to a fever and neck mass that had lasted for 2 days and rash for 1 day. The patient was previously healthy without a history of cardiac or pulmonary disorders, and his family history was unremarkable. Two months before admission, the child showed no signs of SARS-CoV-2 infection and had no history of exposure to COVID-19.Upon initial evaluation, the patient was fully conscious with a body temperature of 36.5°C, a pulse of 116 beats per minute, and a respiratory rate of 22 breaths per minute. His blood pressure was measured at 106/60 mmHg. Auscultation of both lungs revealed normal findings, and physical examinations of the heart and abdomen were also normal.

Prior to admission, he underwent a complete blood count (CBC) and biochemical tests at a secondary hospital. Laboratory examinations revealed a C-reactive protein (CRP) level of 42.26 mg/L, white blood cell count of 13.01×10^9^/L, hemoglobin level of 118 g/L, platelet count of 232×10^9^/L, creatine kinase (CK) of 927 U/L,alanine aminotransferase (ALT) of 219 U/L,aspartate aminotransferase (AST) of 580 U/L, and lactate dehydrogenase (LDH) of 1000 U/L. He was diagnosed with sepsis and acute cervical lymphadenitis and received one day of piperacillin therapy. However, his fever did not show significant improvement.

**Figure 1:**
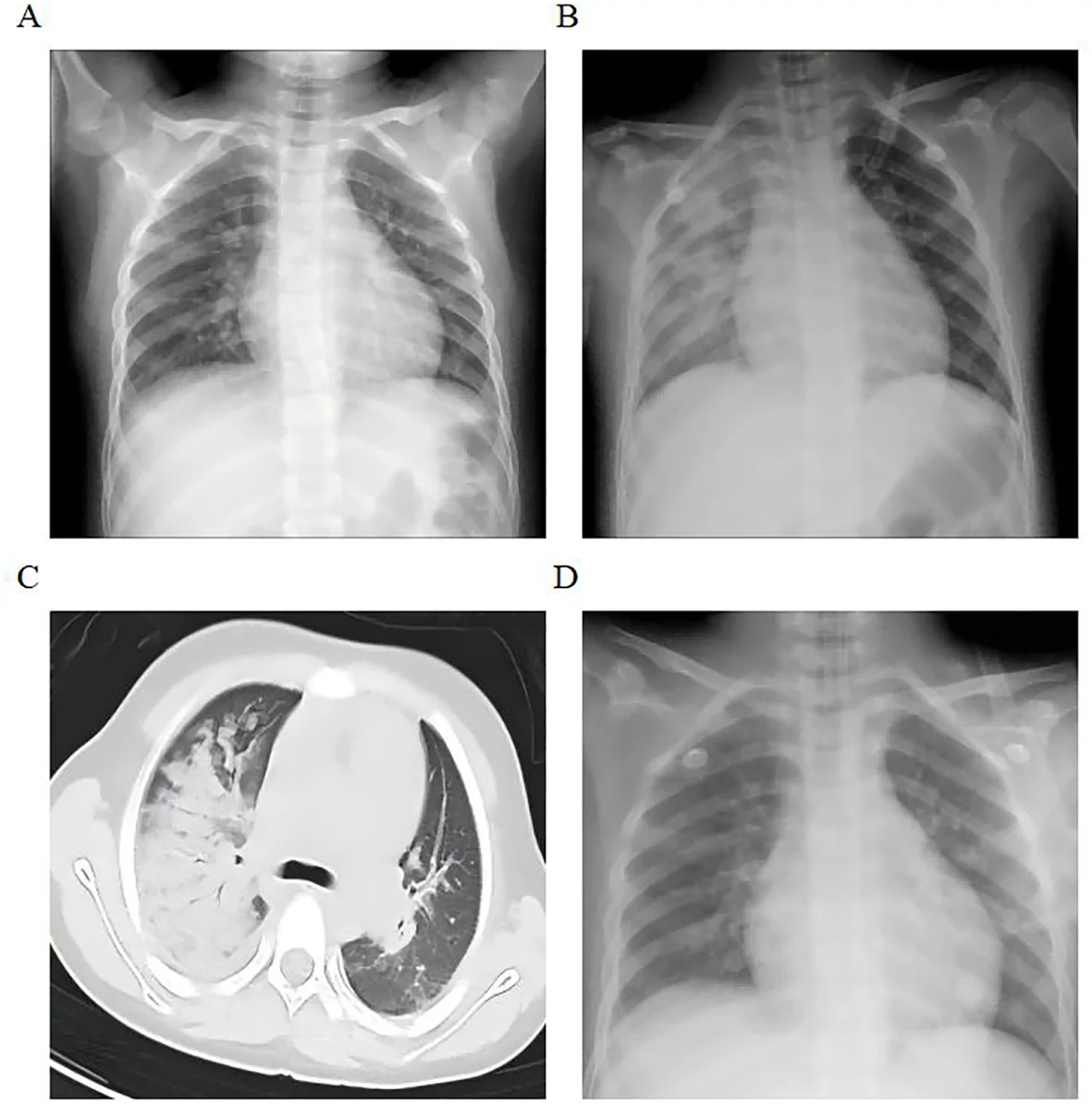
**(A)** The chest X-ray on admission. **(B, C)** The chest X-ray and CT on the day of applying invasive mechanical ventilation. **(D)** The chest X-ray on the day of removing invasive mechanical ventilation.

On admission (day 1), the patient developed high fever with a peak temperature of 41 °C. He presented with rash, strawberry tongue, bilateral conjunctival injection, unilateral cervical lymphadenopathy, and swollen feet. Laboratory tests showed an erythrocyte sedimentation rate (ESR) level of 21 mm/1 hour, procalcitonin of 1.42 ng/mL, creatine kinase isoenzyme of 7.36 µg/L, and normal levels of B-type natriuretic peptide (BNP) and troponin. A chest X-ray showed no pulmonary infiltrates or cardiomegaly (**Figure 1A**). Echocardiography demonstrated normal left ventricular function and no evidence of coronary artery dilation or aneurysm. Blood and urine cultures were negative.

On day 2, the patient developed recurrent fever and was diagnosed with KD based on the diagnostic criteria in the American Heart Association scientific statement (2). He received IVIG (2 g/kg) and aspirin therapy. Considering the possibility of an underlying infection, amoxicillin-clavulanate potassium was also initiated. During the IVIG infusion, the patient suddenly developed dyspnea with oxygen saturation dropping to 85%.Blood pressure at that time was 100/70 mmHg. Auscultation of the right lung revealed crackles, and the liver and spleen were not enlarged. The patient was transferred to the ICU and underwent high-flow nasal cannula (HFNC) oxygen therapy on day 3. However, the patient’s condition continued to deteriorate, and his breathing difficulty worsened. CRP and BNP increased significantly in the patient. Echocardiography showed enlargement of the left atrium and left ventricle, along with reduced left ventricular systolic function, and no signs of coronary artery dilatation. Chest computerized tomography (CT) revealed multiple exudative lesions in both lungs, consolidation in the right lung, and minimal pleural effusion on the right side (**Figures 1B, C**). On day 3, the child underwent bronchoscopy, which revealed acute endobronchitis. Postoperatively, the patient received invasive mechanical ventilation and methylprednisolone(4mg/kg/day).Arterial blood gas analysis and inhaled oxygen concentration were shown in **Table 1**. Given hemodynamic instability, KDSS was confirmed, and intravenous vasoactive drugs were commenced to provide circulatory support. On day 4, sputum culture indicated *Haemophilus influenzae* infection, and the treatment was upgraded to meropenem for anti-infection therapy.

**Table 1:** Arterial blood gas analysis and inhaled oxygen concentration.

Time	PCO2	PO2	FiO2	P/F	Respiratory support
D3	27.1	56.8	0.55	103	IMV
D4	37.5	85.8	0.35	245	IMV
D5	27	147	0.35	420	IMV/HFNC
D6	32	73.9	0.3	246	HFNC
D7	42.4	106	0.41	258	HFNC
D8	39.3	127	0.35	362	HFNC
D9	40.3	102	0.25	408	CNC
D10	38.5	101	0.25	404	CNC

IMV, invasive mechanical ventilation; HFNC, high-flow nasal cannula; CNC, conventional nasal cannula.

On day 5, the patient continued to have a fever for more than 36 hours after the completion of the initial IVIG infusion, and he received a second dose of IVIG therapy. The patient was taken off invasive mechanical ventilation and instead received HFNC on the same day. A chest X-ray was taken and showed complete resolution of pulmonary consolidation and pleural effusion (**Figure 1D**). On day 7, the patient was transferred to the general ward thereafter. Some laboratory parameters during hospitalization were shown in **Table 2**. He was discharged on Day 15 with a low-dose aspirin regimen and oral prednisone.

**Table 2:** Some laboratory parameters during hospitalization.

Time	WBC (10^9^/L)	PLT (10^9^/L)	HB (g/L)	CRP (mg/L)	ESR (mm/1hr)	PCT (ng/ml)	D-dimer (mg/L)	Troponin (ug/L)	BNP (pg/ml)	IL-6 (pg/ml)	IL-8 (pg/ml)	IL-10 (pg/ml)
D0#	13.01	232	118	42.26								
D1					21	1.42		<0.002	32.36			
D2	13.91	237	98	48.35								
D3	14.12	252	86	106.91	83	1.62	1.62	0.1	957.75	117.15	39.79	13.09
D4	18	327	90	101.82								
D5	20.98	370	91	41.78				0.16	505.73			
D6	26.74	449	92	28.09								
D7	30.8	465	94	29.19								
D8	29.34	542	91	26.69		0.16						
D9	24.1	604	97	36.95		0.12						
D13	11.84	873	96	1.25				0.013	52.18			

#D0 was the day prior to admission.

One month after discharge, echocardiography demonstrated normal left ventricular function, and no signs of coronary artery dilatation or pulmonary fibrosis. The patient reported consistent adherence to treatment and proactive engagement with his individualized treatment plan, resulting in globally favorable clinical prognoses. He also expressed intent to fully comply with all medical instructions pertaining to long-term follow-up visits.

## Discussion and conclusion

Here, we describe a typical case of severe KD who developed KDSS and pulmonary involvement that required invasive mechanical ventilation. Multisystem inflammatory syndrome (MIS-C) in children associated with SARS-CoV-2 led to serious and life-threatening illness in previously healthy children (13).While MIS-C was considered in the differential diagnosis given the patient’s presentation, there was no evidence of SARS-CoV-2 infection or prior COVID-19 exposure in this child. In addition, all hallmark clinical manifestations of KD were present, confirming the definitive diagnosis of KD. In 2009, Kanegaye et al. defined the characteristics that distinguish KDSS from hemodynamically normal KD (HNKD) (3). KDSS is a rare but severe form of the illness in which patients experience vasodilatory shock, hypotension, and poor perfusion, with or without myocardial dysfunction (2), which can lead to poor outcomes.

While pulmonary manifestations are not a common feature of KD, they have been reported in approximately 1.83-14.7% of cases (14, 15). In a study of 2,686 pediatric patients diagnosed with KD, 976 underwent chest X-ray or chest CT, and 481 of them showed signs of pulmonary involvement (7).The lung manifestations in patients with KD include pneumonia, pleural effusion, lung consolidation and pulmonary nodules (7, 16, 17). The initial chest X-ray in our patient revealed no pulmonary infiltrates, and he presented no respiratory symptoms upon admission. However, he soon developed dyspnea and required invasive mechanical ventilation.

The pathogenesis of KD has been linked to various infections, including *Streptococcus* (18), *Staphylococcus* (18), *Mycoplasma pneumoniae* (19), SARS-CoV-2 (20, 21), influenza (22) and adenovirus (23). The role of superantigens and cytokines in infection is considered crucial in the pathogenesis of KD (18, 24). However, no definite causal relationship has been established to date. In our case report, the patient’s sputum culture revealed an infection with *Haemophilus influenzae* and he received antibiotic therapy. Therefore, it was important to determine whether the lung consolidation was a result of KDSS or the infection. Nir et al. reported that 41% of patients still had pathological findings on follow-up lung ultrasound after 10–14 days in pediatric patients with community-acquired pneumonia (25). In a study by Alba et al., only 20.8% of patients showed complete resolution on lung ultrasound after one month (26). Most children experience 96% resolution of lung consolidation within 2–3 weeks timeframe (27). In our patient, the consolidation in the right lung was completely absorbed within 2 days, and the course of treatment was much shorter than that of lung consolidation caused by infections.

Considering that lung consolidation appeared during the progression of KDSS in our patient, it was determined that the pathological changes observed in the lung were related to KD. In our case, the consolidation was rapidly absorbed, which further confirmed the diagnosis of KD-related pulmonary involvement.

KD is a systemic vasculitis, and the distribution and incidence of arterial lesions in KD autopsy patients were reported as follows: coronary arteries, 95%; kidney, 75%; lung, 45% (28, 29). Autopsy findings in patients with KD revealed inflammatory infiltrates in the alveolar septa, distributed either diffusely across an entire pulmonary lobe or focally within a lobe (30). The pathophysiological mechanism is hypothesized to involve vasculitis-induced enhancement of vascular permeability (7, 31). Previous studies have shown that the expression of vascular endothelial growth factor was upregulated in blood vessels and organs of patients who died from KD (32). This may increase vascular permeability in various organs including lungs in severe KD patients, leading to edema and inflammation (12). Patients with KDSS tend to produce higher levels of cytokines, which may contribute to the development of systemic capillary leak syndrome (33).Platelet activation plays a central role in the vasculitis process of KD (34). In our patient, there was a significant increase in cytokines, platelets, and CRP, suggesting that the release of inflammatory mediators may have played a crucial role in the development of dyspnea. During the acute phase of KD, excessive platelet activation, endothelial cell injury, and cytokine storm act synergistically, leading to vascular endothelial dysfunction and the formation of a prothrombotic state (35, 36). As seen in previous reports, our patient achieved complete recovery following treatment with IVIG, prednisone and aspirin (12, 37).

Furthermore, it should be highlighted that the patient’s dyspnea worsened during the infusion of IVIG. Therefore, it was essential to determine whether pulmonary involvement occurred secondary to IVIG. Transfusion-related acute lung injury (TRALI) and transfusion-associated circulatory overload (TACO) are life-threatening transfusion adverse reactions resulting in acute respiratory distress and pulmonary edema (38, 39). IVIG may also be associated with TRALI/TACO, which are serious adverse effects of blood transfusion with high mortality (40). IVIG-related TRALI/TACO is rarely reported in children. Judy et al. reported a 2-year-old boy who developed TRALI following IVIG transfusion for KD (41). According to the definition, TRALI/TACO primarily involves bilateral pulmonary edema (42, 43). However, in our patient, chest CT revealed multiple exudative lesions in both lungs, consolidation in the right lung, and minimal pleural effusion on the right side, which differed significantly from previous ones. This makes it difficult to explain the pulmonary involvement in our patients solely by using TRALI/TACO.

This study possesses several inherent limitations. Given its nature as a single-case report, the observations herein cannot be generalized to a wider patient cohort. The lack of control groups and mechanistic studies prevents us from establishing causal relationships between pulmonary involvement and TRALI/TACO.

## Conclusion

Our case report describes the treatment and clinical manifestations of pulmonary involvement secondary to KDSS. KDSS patients may develop dyspnea due to the release of inflammatory mediators. The relationship between pulmonary involvement and TRALI/TACO needs further research. Early aggressive management and comprehensive treatment may improve prognosis.

## Data Availability

The original contributions presented in the study are included in the article/supplementary material. Further inquiries can be directed to the corresponding author/s.
